# The Role of Drug-Coated Balloons in an All-Comer Population: Outcomes from a Two-Center Real-World Registry

**DOI:** 10.3390/medicina62040769

**Published:** 2026-04-16

**Authors:** Florin-Leontin Lazar, Teodor Paul Kacso, Calin Homorodean, Horea-Laurentiu Onea, Ioan-Cornel Bitea, Mihai Ober, Oana Stoia, Minodora Teodoru, Dan-Mircea Olinic

**Affiliations:** 1 4th Department of Internal Medicine, Medical Clinic No. 1, Iuliu Hatieganu University of Medicine and Pharmacy, 40006 Cluj-Napoca, Romania; lazar.leontin@yahoo.com (F.-L.L.); chomorodean@yahoo.com (C.H.); onea.lau@gmail.com (H.-L.O.); danolinic@gmail.com (D.-M.O.); 2 Department of Cardiology, County Clinical Emergency Hospital Sibiu, 550245 Sibiu, Romania; cornelioanbitea@yahoo.com (I.-C.B.); oana.stoia@ulbsibiu.ro (O.S.); minodora.teodoru@ulbsibiu.ro (M.T.); 3 DCB International Academy, 20037 Milan, Italy; 4 2nd Department of Cardiology, County Clinical Emergency Hospital Cluj-Napoca, 400006 Cluj-Napoca, Romania; mihai_ober@yahoo.com; 5 Department of Clinical Medicine, Faculty of Medicine, “Lucian Blaga” University, 550024 Sibiu, Romania

**Keywords:** drug-coated balloons, hybrid, acute coronary syndromes, all-comer

## Abstract

*Background and Objectives:* Drug-coated balloons (DCBs) represent a novel, attractive strategy for coronary revascularization; however, data supporting their use in complex real-world populations remain limited. We aimed to evaluate the safety and efficacy of a DCB-first strategy in a predominantly acute coronary syndrome (ACS) and multivessel disease (MVD) population. *Materials and Methods:* We conducted a prospective two-center observational registry including 115 consecutive patients treated with a DCB-first strategy (DCB-only in 44 patients and a hybrid DCB–drug-eluting stent in 71 patients) for both de novo and in-stent coronary lesions. Bailout stenting was performed when required according to predefined criteria. *Results:* The study population was characterized by high clinical complexity, with 78.3% MVD and 67.8% presenting with ACS, including 10.5% ST-segment elevation myocardial infarctions. Bailout stenting was required in 12.2% of lesions. At 18 months, the target lesion revascularization (TLR) rate was 2.83%, while the device-oriented composite endpoint (DOCE; cardiac death, target vessel myocardial infarction or TLR) occurred in 4.7% of patients. The cumulative major adverse cardiovascular event (MACE) rate at 18 months was 14.8%, largely driven by the high-risk clinical profile of the cohort. Patients treated with a DCB-only strategy had a shorter duration of dual antiplatelet therapy compared with those treated with a hybrid strategy. *Conclusions*: In this two-center real-world registry including predominantly ACS and MVD patients, a DCB-first strategy was associated with low lesion-level event rates and acceptable mid-term clinical outcomes. These findings support the feasibility of a leave-nothing-behind approach in complex coronary disease when meticulous lesion preparation and provisional bailout stenting are applied.

## 1. Introduction

In the past few years, drug-coated balloons (DCBs) have gained impressive popularity in the interventional cardiology arena and have become widely used in multiple coronary artery disease (CAD) scenarios. While the most important trials have focused on the safety and efficacy of this technology in small-vessel disease and in-stent restenosis, recently, complex lesions such as those associated with diffuse disease [1], chronic total occlusions (CTOs) [2] and bifurcation lesions [3], and even acute myocardial infarctions (MIs) [4], have been treated with a stentless strategy in several small studies, with favorable results being reported. Moreover, the combined use of DCBs and drug-eluting stents (DESs) represents an alternative strategy in these complex lesion subsets, offering promising outcomes at long-term follow-up.

The rationale for the use of DCBs for treating these lesions is mainly represented by the proportionally higher rate of stent failure associated with increasing case complexity [5]. Moreover, the lack of a metallic scaffold could provide future surgical alternatives, which is of great interest in younger patients, as well as promoting the restoration of the segment’s vasomotion [5].

Despite its appeal, however, the use of DCBs in these complex lesions should be based on scientific data, which unfortunately are still scarce. In order to shed light on the safety and efficacy of DCBs in de novo complex lesions, data from a large trial comparing sirolimus DCBs and conventional DESs in this setting—the SELUTION DeNovo Trial—were presented during TCT 2025 [6]. As the primary endpoint of target vessel failure (TVF) at 1 year was non-inferior in the DCB group, the question of whether a DCB-based strategy is feasible for an all-comer population has been answered to some extent. However, several scenarios, such as ST-segment elevation MI (STEMI), CTO and in-stent restenosis (ISR), were excluded from this study; as such, further registries and trials that include the full spectrum of pathologies are still needed.

Taking all the above into consideration, the aim of our study was to evaluate the performance of DCBs as a first-line strategy, irrespective of the complexity of the lesion, with bailout stenting when the initial result is unsatisfactory.

## 2. Materials and Methods

### 2.1. Study Design and Patient Population

We conducted a prospective, two-center, investigator-initiated registry evaluating the performance of DCBs in the treatment of a real-world population, including both chronic and ACS (unstable angina, STEMI and N-STEMI). The main purpose of the study was to evaluate whether the use of DCBs as a first-line strategy, irrespective of the complexity of the lesion, with bailout stenting when the final result is considered unsatisfactory, is safe and efficient. For this purpose, between February 2023 and October 2024, we enrolled 115 patients from two tertiary hospitals in Romania, namely the County Clinical Emergency Hospital of Cluj-Napoca and Sibiu. The patients were aged between 33 and 85 years old and presented with either de novo or ISR lesions. Both simple and complex lesions were included, with complex lesions defined as bifurcation lesions, CTO, diffuse lesions, calcific lesions and lesions in patients with a low left-ventricular ejection fraction (LVEF). Operators were encouraged to use a DCB as the first option of treatment after proper lesion preparation and if specific angiographic criteria were met.

The diagnosis of chronic coronary syndrome and ACS was made according to the relevant guidelines [7,8]. Clinical data including demographics, cardiovascular risk factors, history of cardiovascular disease, laboratory data, echocardiographic parameters and medications were collected from the medical charts. The LVEF was estimated using the Simpson biplane method.

The inclusion criteria were represented by (a) age > 18 years and willingness to provide informed consent; (b) significant coronary stenosis. The following exclusion criteria were applied: (1) high thrombotic burden (assessed angiographically according to the Thrombolysis in Myocardial Infarction (TIMI) thrombus grading scale (grade ≥ 4)); (2) spontaneous coronary artery dissection; (3) cardiogenic shock and life expectancy < 1 year.

### 2.2. Study Procedure and Devices

The culprit lesion was identified based on electrocardiographic changes and angiographic appearance. Stenosis severity was assessed both visually and through quantitative coronary analysis, while the highest degree of stenosis was taken into account. A threshold of 70% was used to define a significant coronary lesion. Multivessel disease was defined by an additional significant stenosis in a non-culprit vessel or by the involvement of the left main artery. Coronary flow was evaluated using the TIMI score. Bifurcation lesions were included if the side branch diameter was at least 2 mm. A lesion was classified as diffuse when the length was ≥40 mm. Calcific lesions were classified as complex when moderate to severe calcification was present on angiography (i.e., visible prior to contrast injection).

The procedure was conducted using both paclitaxel–DCB (Essential Pro, iVascular, Barcelona, Spain) and sirolimus–DCB (Sequent SCB, B. Braun, Melsungen, Germany). DCB–percutaneous coronary intervention (PCI) was performed based on expert consensus documents [9,10]. Lesion preparation was therefore mandatory, using a balloon-to-vessel ratio of 1.0/1.0, usually starting with semi- (SC) or non-compliant (NC) balloons. Moreover, the use of specialty balloons (cutting/scoring or high-pressure balloons (OPN)) was encouraged when suitable, as well as intravascular lithotripsy (IVL) or atherectomy in severely calcified lesions. DCB treatment was applied in the presence of the following criteria: TIMI 3 flow, residual stenosis < 30% and non-flow limiting dissection, below type C. Bailout stenting was conversely performed if residual stenosis >30%, as well as in the case of type C or above dissection, TIMI flow < 3 or type A-B dissection with ongoing pain, as deemed necessary by the operator. The DCB was inflated at a balloon-to-vessel ratio of 0.8–1.0/1.0 to the nominal pressure for 30–60 s. The DCB was selected to extend at least 3 mm beyond both edges of the lesion, and, when multiple DCBs were used, overlap was ensured. To account for vessel recoil, the final angiogram was obtained at least 5 min after DCB use.

Bifurcation lesions were treated either with a DCB in both the main and side branches (sequential inflation, kissing optional but highly discouraged due to the risk of dissections) or with a DES in the main branch and a DCB in the side branch (DCB inflation preferably performed prior to stenting). Diffuse lesions were managed using either a DCB-only strategy or a combination of a DES and DCB. A hybrid strategy was defined as the use of both a DCB and a DES either within the same lesion (e.g., long diffuse lesions treated with a DCB distally and a DES proximally) or in patients with additional lesions (in the same or different vessels) requiring stent implantation after treatment of the culprit lesion with a DCB, or vice versa. We do not use this term for patients who required bailout stenting.

All patients were pretreated with acetylsalicylic acid 300 mg and clopidogrel 300/600 mg or ticagrelor 180 mg as loading doses, followed by intravenous heparin to maintain an activated clotting time of >250 s during the procedure. Dual antiplatelet therapy (DAPT) was recommended for at least 1 month after the procedure.

### 2.3. Study Endpoints

The study’s primary endpoint was target lesion revascularization (TLR) at 18-month follow-up. Secondary clinical endpoints were major adverse clinical events (MACE) throughout 18 months of follow-up and device-oriented composite endpoints (DOCE) at 18 months. MACE were defined as a composite of death of any causes, MI, heart failure requiring hospitalization, TLR, target vessel revascularization (TVR) and major bleeding. As repeated hospitalization and major bleeding might have influenced the results, we decided to perform a separate analysis of DOCE. The individual components of DOCE were cardiac death, TV-MI and TLR. TLR was defined as repeated PCI or coronary artery bypass grafting in the target segment, including 5 mm proximal and distal to the previously treated lesion. TVR was defined as any repeat PCI or coronary bypass of any segment of the target vessel, including the target lesion. Major bleeding events were defined as types 3–5 according to the Bleeding Academic Research Consortium classification.

Follow-up was performed through ambulatory visits or telephone contact, while, for cases in which patients could not be contacted, we obtained survival registries from the local authorities. Our study protocol was carried out according to the Declaration of Helsinki for human research and was endorsed by the University of Medicine and the Pharmacy “Iuliu Hatieganu” Clinical Research Ethics Committee (AVZ 199, on 26 July 2023) and the County Emergency Hospital Sibiu Clinical Research Ethics Committee (No. 2995, on 9 February 2023).

### 2.4. Statistical Analysis

Statistical analyses were carried out using R and R studio v 2025.05.1 (Posit, PBC, MA, USA). Normal distributions were assessed using descriptive measures of skewness and kurtosis, as well as the Shapiro–Wilk test. Group comparisons were performed using the chi-squared test for categorical variables; for continuous variables, we used Student’s *t*-test for independent samples or the Mann–Whitney U test in the case of non-parametric variables. Parametric variables are presented as the mean ± S.D. and non-parametric variables as the median (IQ range). Survival analysis was conducted using Kaplan–Meier curves, and differences between groups were assessed with log-rank values. A *p*-value of less than 0.05 was considered statistically significant.

## 3. Results

### 3.1. Baseline Clinical and Procedural Characteristics

**Figure 1:**
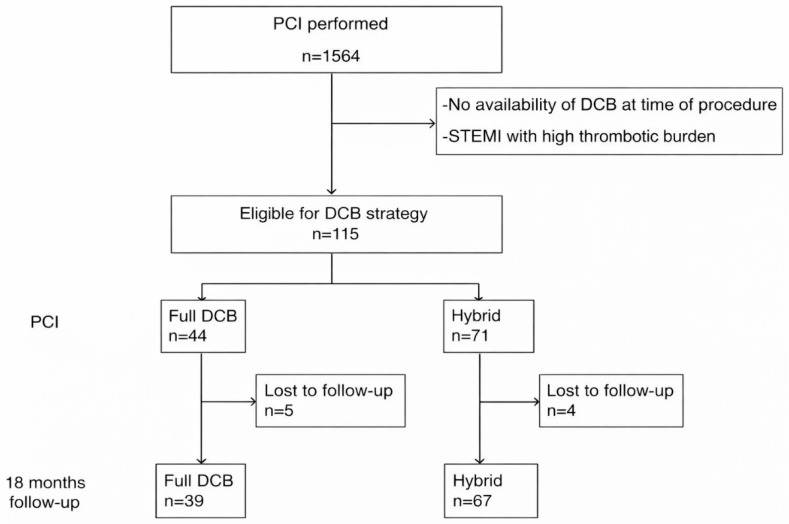
Flowchart of patient selection.

Between February 2023 and October 2024, we performed 1564 coronary interventions in two county emergency hospitals in Transylvania, Romania. Due to various periods of unavailability of DCBs, we were not able to enroll all consecutive patients who would have been eligible for the study. Moreover, as both centers were tertiary centers devoted mainly to ACS, a significant proportion of the sample was represented by STEMI patients with a high thrombotic burden or cardiogenic shock, who were also not eligible for the study. As a result, we enrolled 115 patients for whom a DCB represented the first line of interventional treatment, irrespective of lesion complexity, 44 of whom were treated with a full-DCB approach. Figure 1 shows the study composition and flowchart.

Out of the 115 patients, 73% were men, 52.2% had type 2 diabetes and 60% had a previous PCI with stent implantation. Of note, 64.3% of patients had de novo lesions and the vast majority presented with ACS (29.5% unstable angina, 27.8% NSTEMI and 10.5% STEMI). The main baseline characteristics are summarized in Table 1.

A full-DCB strategy was used in 37% of cases, while a hybrid strategy (meaning the use of a DES for the same lesion or additional lesions within the same or different vessels) was used in 63% of cases. There were no significant differences between the two groups (Table 2).

All patients benefited from aggressive lesion preparation, using different types of balloons (SC, NC, OPN), IVL or rotational atherectomy. Of note, a significant number of lesions were ostial (34.7%) or bifurcation lesions (47.8%), and 73.9% of patients had large vessels, treated with DCBs as the first line. Most of the patients had multivessel disease (MVD; 78.3%). Imaging was used in only 12.2% of cases; however, angiographic follow-up was performed in 20% of cases. Procedural success was achieved in all patients, but with 12.2% bailout stenting in the entire cohort, mainly for important recoil or dissections. Table 3 summarizes the main procedural characteristics.

### 3.2. Primary and Secondary Endpoints

**Figure 2:**
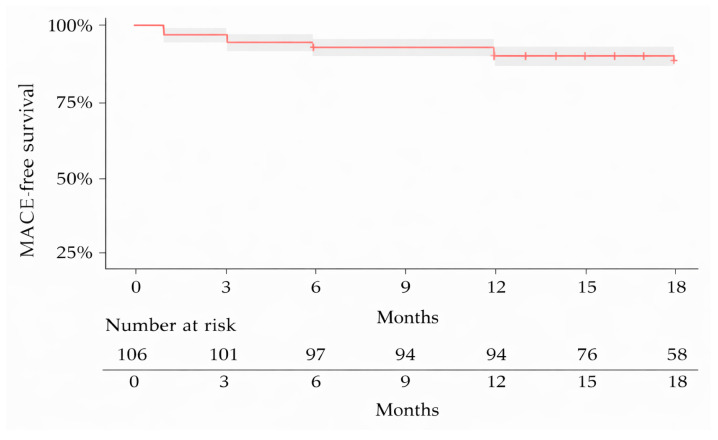
Kaplan–Meier estimates of MACE-free survival at 18 months.

Follow-up was mainly conducted through ambulatory visits and, in some cases, telephonically. During the ambulatory visits, if considered necessary, patients were scheduled for angiographic follow-up (not only symptom-related, but also for very complex lesions or dissections left untreated). Nine patients were lost to follow-up and three patients died during the follow-up period, with none of the deaths being device-related (one due to metastatic pulmonary cancer, one during non-cardiac surgery, and one due to severe renal failure). Follow-up information was therefore available for 106 patients (92.1%). TLR occurred in three patients (2.83%). The incidence of MACE was as follows: at 1 month—4.7%, at 3 months—8.5%, at 6 months—9.4%, at 12 months—13.3% and at 18 months—14.8% (Figure 2). It should be noted that, at each time point, MACE represented the cumulative total of previously recorded events plus any new events occurring during the respective period.

Of note, the DAPT duration was reduced in patients treated with a DCB-only strategy versus those treated by a hybrid strategy, with a mean difference of 1.9 months of DAPT (95% CI: −3.6 to −0.1, *p* = 0.037), while the length of hospitalization did not differ between groups (2.63 vs. 2.89 days, *p* = 0.41).

**Figure 3:**
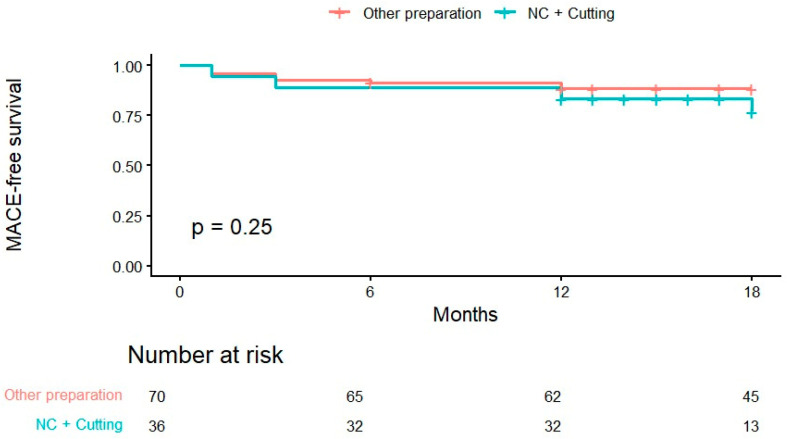
Kaplan–Meier estimates of MACE-free survival according to lesion preparation strategy.

Sequential lesion preparation using SC, NC and cutting balloons was required in 33.9% of cases due to significant calcification. As adequate lesion preparation is a cornerstone of DCB-PCI, we therefore compared this subgroup of patients in which aggressive preparation was performed with the remainder of the cohort to evaluate whether intensified lesion preparation influenced clinical outcomes. At 18 months, Kaplan–Meier estimated MACE-free survival was 88.5% (95% CI 81.3–96.3%) in the non-NC + cutting group and 76.9% (95% CI 62.1–95.3%) in the NC + cutting group (Figure 3). The difference was not statistically significant (log-rank *p* = 0.25). Cox regression analysis showed a non-significant trend toward a higher risk in the NC + cutting group (HR 1.79, 95% CI 0.65–4.95; *p* = 0.25).

MACE rates were subsequently compared between patients managed with a DCB-only strategy and those requiring adjunctive DES implantation, i.e., the hybrid group. At 18 months, the Kaplan–Meier-estimated MACE rates were 19.4% in the full-DCB group and 11.9% in the hybrid group (log-rank *p* = 0.39). As the inclusion of major bleeding and heart failure hospitalization within the composite MACE value may introduce heterogeneity and potentially influence the comparison between treatment strategies, we further analyzed the individual components of MACE (Table 4).

**Figure 4:**
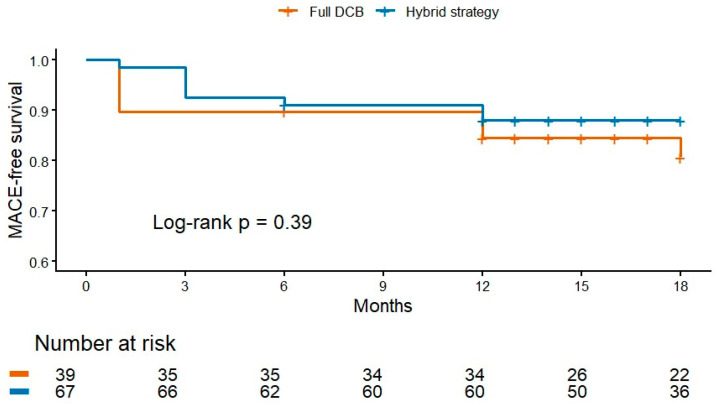
Kaplan–Meier estimates of MACE-free survival according to treatment strategy (DCB-only vs. DCB + adjunctive DES).

In a univariable Cox regression analysis, the hybrid strategy was not associated with a significant difference in MACE compared with full DCB (HR 0.64, 95% CI 0.23–1.76; *p* = 0.39) (Figure 4).

**Figure 5:**
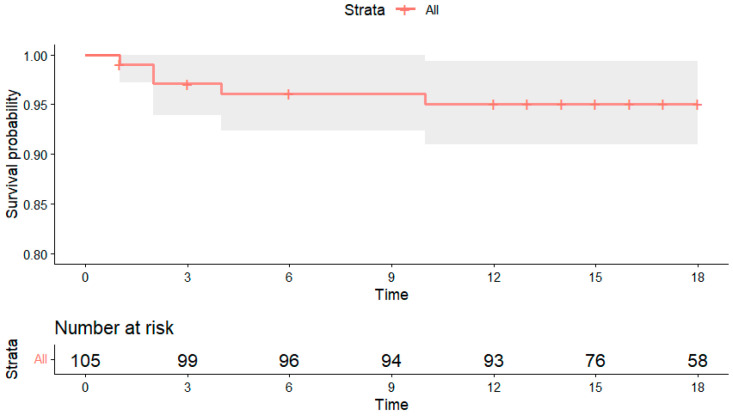
Kaplan–Meier estimates of DOCE at 18 months.

As the population included an important number of ACS patients with a low LVEF, with a high probability of further hospitalization for heart failure, we decided to further investigate the incidence of DOCE. During the 18-month follow-up, DOCE occurred in five patients. The individual components of DOCE were one cardiac death, one TV-MI and three TLR events. The Kaplan–Meier-estimated DOCE-free survival at 18 months was 95.1% (95% CI 90.9–99.4), corresponding to a DOCE incidence of 4.9% (Figure 5).

## 4. Discussion

Complex coronary lesions and patients represented by calcific, diffuse or bifurcation lesions, CTO, ACS and high bleeding risks, as well as MVD patients, are still at risk for long-term device failure, even with the current generation of DESs [5]. In these complex subsets, a DES approach has failed to demonstrate non-inferiority to coronary artery bypass grafting (CABG) with respect to the cumulative incidence of clinical events during short-term follow-up. Furthermore, long-term data indicate that DESs are associated with a higher risk of TVR compared with CABG [11]. These findings highlight the limitations of permanent metallic implants, particularly in patients with a complex coronary anatomy and MVD.

Consequently, DCBs, aiming to minimize permanent metal implantation, have emerged as an attractive strategy, as multiple studies have suggested that a greater stent burden is associated with worse long-term clinical outcomes [12]. While DCBs have demonstrated efficacy in ISR and small-vessel disease [13,14,15,16], their role as a first-line strategy in unselected populations with high lesion complexity, including ACS, remains insufficiently explored. Furthermore, several studies have yielded conflicting results, largely attributable to variations in DCB use protocols and inconsistencies in study design.

For example, in the REC-CAGEFREE I trial [17], a DCB as a first-line strategy with bailout stenting failed to achieve non-inferiority versus routine thin-strut sirolimus-eluting stent implantation in terms of DOCE at 3 years (8.2% vs. 5.0%; difference: 3.21%; 95% CI: 1.17–5.26%; *p* = 0.002)). This large, multicenter study included 2272 patients with non-complex de novo lesions treated across 43 centers in China.

These findings highlight the challenges associated with the broader application of DCBs in de novo lesions. However, differences in study design, patient selection and device platforms may have contributed to these results. In particular, the use of specific DCB technologies not widely available in routine clinical practice, as well as the inclusion of predominantly non-complex lesions, may limit the generalizability of these findings to more heterogeneous, real-world populations [18,19].

Accordingly, the present study sought to evaluate two well-established and clinically validated DCB platforms (Essential Pro, iVascular, Spain and SeQuent SCB, B. Braun, Germany) as a first-line treatment strategy in a highly complex population predominantly comprising patients with ACS and MVD, with provisional bailout stenting when required.

The main findings were as follows:

- Optimal lesion preparation followed by a DCB-first strategy, with provisional bailout stenting when indicated, yielded a low 18-month TLR rate (2.83%) in complex coronary lesions.
- In this clinically complex population, the cumulative 18-month MACE rate was 14.8%, which may be considered acceptable given the inclusion of all-cause death, MI, heart failure hospitalization, revascularization and major bleeding.
- The 18-month DOCE rate was 4.7%, suggesting favorable device-oriented outcomes and that most adverse events were not directly attributable to the treated lesion or the DCB strategy, particularly in a population predominantly presenting with ACS and subsequent recurrent hospitalizations for heart failure.
- In this complex population, bailout stenting occurred in 12.2% of cases despite meticulous lesion preparation.

At the outset, the substantial complexity of the study population should be highlighted, as 78.3% of patients had MVD and 38.3% presented with acute MI, of whom 10.5% had STEMI. As a comparison, in the SELUTION DeNovo Trial, patients with STEMI or NSTEMI with ongoing pain, CTO, ISR or <30% LVEF were excluded [6]. In a similar fashion, in the REC-CAGEFREE I trial [17], patients requiring complex PCI (including atherectomy devices, three-vessel disease, left main lesions, CTO) were excluded. Consequently, a more aggressive lesion preparation strategy was adopted in our cohort, with the sequential use of SC, NC and cutting balloons in 33.9% of patients. As previously reported, the use of calcium-modifying balloons has been associated with favorable clinical outcomes, particularly in de novo lesions treated without additional stent implantation [20]. In our cohort, the consistent use of comprehensive lesion preparation may have contributed to the low 18-month TLR (2.83%) and DOCE (4.7%) rates observed, despite the high prevalence of ACS and MVD.

Our findings are broadly consistent with prior reports in the literature. In the SELUTION DeNovo Trial, which analyzed a cohort of complex patients, TVF was 5.3% at 1-year follow-up, mainly driven by a 3.3% rate of TVR, and it was non-inferior to a DES strategy (risk difference: 0.91%, upper two-sided 95% CI: 2.38%, non-inferiority margin: 2.44%, *p* = 0.02) [6]. Our cohort consisted of 67.8% ACS patients—a subset of high-risk patients for whom data on the use of DCBs are still limited. Recent registry data suggest that, after adjustment for baseline and treatment-related factors, 1-year outcomes are largely similar between STEMI and NSTEMI patients. Accordingly, the MACE rate observed in our study likely reflects the overall clinical risk and revascularization complexity of the ACS population, rather than the infarct type alone [21]. In the PEPCAD NSTEMI trial [22], which enrolled 210 NSTEMI patients (64.2% with MVD), DCB treatment was non-inferior to stent implantation, with a target lesion failure (TLF) rate of 3.8% compared with 6.6% in the stent group. Moreover, MACE occurred numerically less frequently in the DCB arm (6.7% vs 14.2%), supporting the feasibility of a stentless strategy in acute MI. Similarly, a large meta-analysis including a comparable proportion of ACS patients (≈67%) found no significant difference in TLR between DCB and DES strategies (4.3% vs. 6.9%; OR 0.71, 95% CI 0.5–1.0, *p* = 0.059), while TLF was lower in the former (6.1% vs. 16.0%, OR 0.37, 95% CI 0.22–0.59, *p*  < 0.001), over a pooled follow-up period of approximately 26 months [4]. The particularly low TLR rate observed in our study may be explained by the systematic and aggressive lesion preparation strategy adopted. Although extensive predilatation may raise concerns in the ACS setting, plaque-modifying techniques prior to DCB deployment have been shown to be safe and potentially beneficial. In a single-center study by Merinopoulos et al., the use of scoring balloons during lesion preparation in STEMI patients treated with a DCB-only strategy was associated with lower TLR compared with conventional preparation (0.8% vs. 4.2%), supporting the importance of adequate lesion preparation for optimal DCB outcomes [23].

Regarding large-vessel disease, which represented 72.2% of our patients, multiple recent studies have reported encouraging results. In a multicenter, prospective, observational study enrolling 119 patients with de novo coronary lesions in vessels with a reference vessel diameter of 3.1 ± 0.3 mm, TLF occurred in five (4.2%), TLR in four (3.4%) and TVR in five (4.2%) cases, suggesting the safety of this strategy in large vessels [24]. Similar observations were reported by Leone et al., who evaluated the safety and efficacy of a DCB-based strategy in large coronary vessels (>3.0 mm) with de novo lesions in a retrospective cohort of 93 patients (100 lesions). During a mean follow-up period of approximately 350 days, the rate of TVR was 6.6%, with no cases of definite or probable acute vessel occlusion. Despite a relatively long mean lesion length (45 ± 26 mm) and large vessel diameter (3.2 ± 0.3 mm), a DCB-only approach was feasible in 70% of lesions, with bailout stenting required in only 6% of cases and a hybrid strategy adopted in the remainder. These findings highlight the potential advantages of DCB-based strategies in large vessels, as minimizing permanent metal implantation may reduce the total stent length and potentially allow shorter DAPT while preserving vessel physiology [25]. In our study, similarly favorable lesion-level outcomes were observed, with a TLR rate of 2.83% and an overall DOCE rate of 4.7% at 18 months, despite the higher clinical complexity of our cohort, characterized by a high prevalence of ACS and MVD. Moreover, a numerical trend towards fewer DOCE events was observed when comparing the hybrid vs. full-DCB groups; however, this finding should be interpreted with caution given the low number of events and the limited statistical power of the study. The overall 18-month MACE rate of 14.8% appears acceptable considering the high-risk clinical profile of the population, including recurrent hospitalizations for heart failure. These findings further support the feasibility of a DCB-first strategy in complex real-world settings.

In this context, DOCE may represent a more appropriate endpoint for evaluating the performance of DCB-based strategies, as it better reflects device-related outcomes compared with broader composite endpoints such as MACE.

An additional relevant finding of the present study was the reduction in the DAPT duration among patients treated with a DCB-only strategy compared with those requiring adjunctive DES implantation. The mean difference was approximately 1.9 months (95% CI: –3.6 to –0.1; *p* = 0.037). As bleeding after PCI has been identified as a strong independent predictor for 1-year mortality in several reports [26], strategies aimed at minimizing the bleeding risk are of particular clinical relevance. Current expert consensus documents support a shortened DAPT duration of approximately four weeks following DCB treatment of de novo lesions [9,10]. In this context, our findings further support the theoretical advantage of a leave-nothing-behind strategy, which may allow a safer reduction in DAPT exposure while maintaining favorable clinical outcomes.

In contrast, the length of hospitalization did not differ significantly between strategies (2.63 vs. 2.89 days; *p* = 0.41). This was not unexpected given the high-risk profile of our cohort, characterized by a substantial proportion of ACS patients and reduced LVEFs.

While bailout stenting has been shown to represent an independent risk factor for TVF at 1 year in the BOSS study [27], where bailout stenting occurred in a similar proportion of patients (11.1%) compared with our cohort (12.2%), TVF was observed in only 2.9% of patients in our study. This discrepancy may be explained, at least in part, by the larger reference vessel diameter in our population (2.97 mm vs. 2.5 mm in the BOSS study), as small-vessel disease has consistently been shown to represent an independent predictor of device failure [28,29].

Moreover, the systematic and aggressive lesion preparation strategy employed prior to definitive treatment may have further mitigated the potential adverse impacts of bailout stenting.

Several limitations of the present study should be acknowledged. First, this was a non-randomized, observational registry without a control group treated with a contemporary DES, limiting the ability to draw causal inferences or directly compare outcomes. Second, the sample size was low, and, although reflective of real-world practice, combined with the low number of events, it represents an important limitation when interpreting the results and identifying meaningful differences between groups. Third, patient selection may have been subject to bias, as patients were enrolled from high-volume STEMI centers, where operators were encouraged to adopt a DCB-first strategy when deemed feasible. However, a substantial proportion of patients were not included due to clinical or hemodynamic instability, particularly in acute settings, where rapid and predictable revascularization was required. In these cases, operators often favored immediate stent implantation to ensure prompt vessel stabilization, even when lesions might have been technically suitable for a DCB-based approach. This reflects real-world practice in ACS, where time constraints and patient instability may limit the applicability of more preparation-dependent strategies such as DCB-PCI. Fourth, procedural decisions, including the lesion preparation intensity and bailout stenting thresholds, were left to operator discretion and may have introduced treatment bias. Moreover, another limitation of this study was the lack of a uniform revascularization strategy across all lesions, as comparisons between DCB-only and hybrid approaches may be confounded by the treatment of additional lesions. This is particularly relevant in an all-comer population, where complete revascularization often requires a combination of treatment modalities tailored to the lesion characteristics. Fifth, although follow-up was extended to 18 months for clinical outcomes, longer-term data are not yet available, and late divergence in event rates cannot be excluded. Sixth, the use of intracoronary imaging was low, due to local resource availability. While we acknowledge that the use of optical coherence tomography or intravascular ultrasound (IVUS) might have yielded more optimal results, potentially leading to different treatment decisions, the recently published IVUS-CHIP trial [30], which evaluated IVUS-guided vs. angiography-guided PCI in complex high-risk patients, did not demonstrate a significant reduction in MACE despite improved procedural metrics. Seventh, a multivariable analysis was considered. However, given the limited number of events in our cohort (15 MACE events and three TLR events among 106 patients with available follow-up), a multivariable Cox model would not have been statistically robust. In accordance with established recommendations regarding events per variable in regression analyses, the number of events was insufficient to allow reliable adjustment for potential confounders without a substantial risk of overfitting and unstable estimates. Therefore, we restricted our analysis to univariable models, and these results should be interpreted as exploratory. Finally, angiographic follow-up was performed in a subset of patients (20%) based on clinical or procedural indications, which may have exposed these patients to procedure-related risks and potential bias.

## 5. Conclusions

In this two-center real-world registry of predominantly ACS and MVD patients, a DCB-first strategy (full-DCB or hybrid DCB-DES) was associated with low device-oriented event rates and acceptable mid-term clinical outcomes. Despite the inherent limitations of the study design, these findings support the feasibility of a leave-nothing-behind approach in complex coronary disease when meticulous lesion preparation and bailout stenting are applied. Larger prospective studies and randomized controlled trials are warranted to confirm these results.

## Figures and Tables

**Table 1:** Baseline characteristics.

Number of patients (n (%))	115
Age (years)	65.9 ± 10.1
Male sex	73 (63.5%)
Diabetes	60 (52.2%)
Hypertension	98 (85.2%)
Prior MI	57 (49.5%)
Prior PCI	60 (69%)
ISR	41 (35.7%)
De novo lesions	74 (64.3%)
Stable CAD	37 (32.2%)
Unstable angina	34 (29.5%)
NSTEMI	32 (27.8%)
STEMI	12 (10.5%)
LVEF (%)	47.5 ± 9.07
Creatinine (mg/dL)	0.98 (0.81–1.20)

Values are mean ± S.D. or median (interquartile range), unless otherwise indicated. CAD: coronary artery disease; ISR: in-stent restenosis; LVEF: left-ventricular ejection fraction; MI: myocardial infarction; NSTEMI: non-ST-segment elevation MI; PCI: percutaneous coronary intervention; STEMI: ST-segment elevation MI.

**Table 2:** DCB-only vs. hybrid strategy baseline characteristics.

Variable	DCB-Only (n = 44)	Hybrid (n = 71)	*p*-Value
Age (years)	66.9 ± 11.1	65.8 ± 9.8	0.586
LVEF (%)	47.8 ± 8.5	47.2 ± 9.6	0.699
Creatinine (mg/dL)	0.99 (0.89–1.19)	0.96 (0.80–1.29)	0.397
Male sex	28 (63.7%)	45 (63.3%)	0.660
Diabetes mellitus	20 (45.5%)	40 (56.3%)	0.562
Previous MI	22 (50.0%)	35 (49.2%)	1.000
Previous PCI	25 (56.8%)	35 (49.3%)	0.167
ISR	19 (43.2%)	22 (31.0%)	0.137
De novo lesions	25 (56.8%)	49 (69.0%)	0.456
Stable CAD	14 (31.8%)	23 (32.3%)	1.000
Unstable angina	11 (28.9%)	20 (29.0%)	1.000
NSTEMI	12 (31.6%)	20 (29.0%)	0.952
STEMI	4 (9.1%)	8 (11.3%)	0.972

Values are mean ± S.D. or median (interquartile range), unless otherwise indicated. CAD: coronary artery disease; ISR: in-stent restenosis; LVEF: left-ventricular ejection fraction; MI: myocardial infarction; NSTEMI: non-ST-segment elevation MI; PCI: percutaneous coronary intervention; STEMI: ST-segment elevation MI.

**Table 3:** Procedural characteristics.

LESION PREPARATION	
SC BALLOON	111 (96.52%)
NC BALLOON	80 (69.56%)
CUTTING BALLOON	41 (35.65%)
OPN	4 (3.47%)
IVL	3 (2.60%)
ROTABLATION	1 (0.80%)
LESION LENGTH (mean, mm)	24.96
VESSEL DIAMETER (mean, mm)	2.94
OSTIAL LESIONS (%)	40 (34.7%)
BIFURCATION LESIONS (%)	55 (47.8%)
LAD-LCx (%)	20 (36.3%)
LAD-D (%)	16 (29.02%)
LCX-OM (%)	13 (23.65%)
PDA-PL (%)	6 (10.9%)
SMALL-VESSEL DISEASE (%)	30 (26.1%)
LARGE-VESSEL DISEASE (%)	85 (73.9%)
CTO (%)	3 (2.7%)
MULTIVESSEL DISEASE (%)	90 (78.3%)
IMAGING USE (%)	14 (12.2%)
ANGIOGRAPHIC FOLLOW-UP (%)	23 (20%)
BAILOUT STENTING (%)	14 (12.2%)

Values are mean ± S.D. or median (interquartile range), unless otherwise indicated. CTO: chronic total occlusion; D: diagonal branch; IVL: intravascular lithotripsy; LAD: left anterior descending artery; LCx: left circumflex artery; NC: non-compliant; OM: obtuse marginal branch; OPN: high-pressure balloon; PDA: posterior descending artery; PL: posterolateral artery; SC: semi-compliant.

**Table 4:** Individual components of MACE between groups.

Component	DCB-Only (n = 39)	Hybrid (n = 67)
Unstable angina	2 (5.3%)	2 (3.0%)
Myocardial infarction	0 (0.0%)	1 (1.5%)
Heart failure hospitalization	1 (2.6%)	3 (4.5%)
TLR	3 (7.7%)	0 (0.0%)
TVR	1 (2.6%)	0 (0.0%)
Death (all-cause)	1 (2.6%)	1 (1.5%)
Major bleeding	0 (0.0%)	2 (3.0%)

TLR: target-lesion revascularization; TVR: target-vessel revascularization.

## Data Availability

The data presented in this study are available on request from the corresponding author. The data are not publicly available because they are the property of Sibiu County Emergency Hospital, Sibiu, Romania.

## Abbreviations

The following abbreviations are used in this manuscript:

ACS	Acute coronary syndromes
CABG	Coronary artery bypass grafting
CAD	Coronary artery disease
CTO	Chronic total occlusion
DAPT	Dual antiplatelet therapy
DCB	Drug-coated balloon
DES	Drug-eluting stent
DOCE	Device-oriented composite endpoints
ISR	In-stent restenosis
IVL	Intravascular lithotripsy
LVEF	Left-ventricular ejection fraction
MACE	Major adverse cardiovascular events
MVD	Multivessel disease
NC	Non-compliant balloon
OPN	High-pressure balloon
PCI	Percutaneous coronary intervention
STEMI	ST-segment elevation myocardial infarction
TLF	Target lesion failure
TLR	Target lesion revascularization
TVF	Target vessel failure
TVR	Target vessel revascularization
